# Advances in Bioprocess Engineering for Optimising *Chlorella vulgaris* Fermentation: Biotechnological Innovations and Applications

**DOI:** 10.3390/foods13244154

**Published:** 2024-12-22

**Authors:** Ana R. Mendes, Maria P. Spínola, Madalena Lordelo, José A. M. Prates

**Affiliations:** 1 CIISA—Centro de Investigação Interdisciplinar em Sanidade Animal, Faculdade de Medicina Veterinária, Universidade de Lisboa, Av. da Universidade Técnica, 1300-477 Lisboa, Portugal; rmendes@isa.ulisboa.pt (A.R.M.); mariaspinola@fmv.ulisboa.pt (M.P.S.); 2 Associate Laboratory for Animal and Veterinary Sciences (AL4AnimalS), Av. da Universidade Técnica, 1300-477 Lisboa, Portugal; 3 LEAF—Linking Landscape, Environment, Agriculture and Food Research Centre, Instituto Superior de Agronomia, Universidade de Lisboa, Tapada da Ajuda, 1349-017 Lisboa, Portugal; mlordelo@isa.ulisboa.pt; 4 Associate Laboratory TERRA, Instituto Superior de Agronomia, Universidade de Lisboa, Tapada da Ajuda, 1349-017 Lisboa, Portugal

**Keywords:** *Chlorella vulgaris*, microalgae fermentation, bioprocess optimization, strain improvement, bioreactors, biopharmaceuticals, bioactive compounds, functional foods

## Abstract

*Chlorella vulgaris*, a unicellular green microalga, has obtained significant attention due to its high protein content, abundance of bioactive compounds, and broad biotechnological potential. Used in nutraceuticals, pharmaceuticals, and functional foods, it is now gaining traction in cosmetics, biopharmaceuticals, and environmental applications. Recent advancements in fermentation technology, such as the development of high-density fermentation strategies, adaptive evolution of strains, and real-time monitoring systems, have greatly improved the efficiency, scalability, and sustainability of *C. vulgaris* production, enhancing bioavailability and product quality. This review explores developments in *C. vulgaris* fermentation, highlighting advancements in strain improvement through genetic engineering, metabolic optimization, mutagenesis, and adaptive evolution, alongside bioprocess engineering and the optimization of fermentation parameters. Key considerations include bioreactor design, downstream processing, and innovative monitoring technologies aimed at maximizing biomass yield and bioactive compound production. Emerging applications of fermented *C. vulgaris* across industries are also highlighted, along with future perspectives on scaling up production, addressing regulatory challenges, and ensuring biosafety. These insights provide a comprehensive outlook on the future of *C. vulgaris* fermentation in biotechnological applications.

## 1. Introduction

The unicellular green microalga *Chlorella vulgaris* (*C. vulgaris*) has become widely recognized for its high nutritional profile and significant biotechnological potential. It is a rich source of proteins, polyunsaturated fatty acids (PUFAs), vitamins (such as B12 and C), and various bioactive compounds, including carotenoids, chlorophylls, and antioxidants. These attributes position *C. vulgaris* as a promising dietary supplement and a valuable ingredient for functional foods [1]. Additionally, it plays an important role in industries focused on renewable energy, bioremediation, and nutraceuticals, highlighting its versatility in various applications [2]. The potential for *C. vulgaris* to serve as a bioinoculant in sustainable agriculture further illustrates its multifaceted applications [3], from enhancing soil fertility to supporting environmentally friendly practises, underscoring its versatility and value across different sectors.

Economically, *C. vulgaris* offers significant value in biotechnology due to its low production costs, sustainable cultivation in systems like photobioreactors, and ability to thrive in diverse environments. In addition, its capacity for carbon dioxide fixation makes it an essential solution for carbon sequestration [4]. With its expanding applications in biofuels, food supplements, and wastewater treatment, *C. vulgaris* is set to maintain a key role in addressing both nutritional and environmental challenges [1,2]. Reflecting this growing importance, the global market for *Chlorella* is experiencing significant growth, valued at approximately USD 0.3 billion in 2022 and projected to grow from USD 0.32 billion in 2023 to USD 0.52 billion by 2030, with a compound annual growth rate of 8.4% during the forecast period (2023–2030) [5]. This growth is primarily driven by the rising demand for natural, plant-based supplements, functional foods, and biofuels, with the Asia–Pacific region, particularly countries like Japan and China, leading due to high consumer awareness and advanced cultivation technologies [6,7].

Due to its rich nutritional content, *C. vulgaris* is widely used in the nutraceutical sector as a dietary supplement in powder or tablet form. It is particularly valued for its high protein content, vitamins, and antioxidants, which are essential for immune function and overall health [8]. The pharmaceutical industry is exploring *C. vulgaris* for its bioactive compounds, which exhibit anti-inflammatory, antioxidant, and immunomodulatory properties [1,7]. Moreover, its antioxidant properties make it a popular ingredient in cosmetic products, where it helps protect the skin from oxidative stress and promotes skin health [2].

In the food and beverage industries, *C. vulgaris* is increasingly used to enhance the nutritional value of products such as health drinks and protein bars. Its digestibility and high levels of essential nutrients make it a valuable supplement for human and animal nutrition [7]. Beyond its nutritional benefits, *C. vulgaris* also plays a role in sustainable development through its potential use in biofuel production and wastewater treatment, demonstrating its environmental contributions [1,9]. The cultivation of this microalga in various conditions, including wastewater treatment systems, not only helps with bioremediation but also produces biomass for nutraceutical applications [10]. This dual functionality aligns with the growing consumer demand for sustainable and eco-friendly food sources, further positioning *C. vulgaris* as a key player in the future of functional foods. It also aligns with the 2023 Agenda for the United Nations Sustainable Development Goals.

Building on these sustainable practises, fermentation has emerged as a critical technique to further enhance the bioactivity, nutrient availability, and economic viability of *C. vulgaris* production. Through microbial fermentation, nutrients such as amino acids and vitamins become more bioavailable, while the process also breaks down the rigid cell walls of *C. vulgaris*, facilitating the release of bioactive compounds [7]. This technique improves nutrient absorption, enhances flavour, and reduces undesirable odours, making it more suitable for various food and industrial applications [2].

On an industrial scale, fermentation allows for more efficient biomass production. Optimized bioreactor designs and process control systems enable the scalable production of *C. vulgaris* with consistent quality and high yields. Furthermore, integrating *Chlorella* into biorefinery processes, such as the simultaneous production of bioethanol and biopeptides, underscores its biotechnological versatility and economic potential [1].

This review provides a comprehensive evaluation of the advances in *C. vulgaris* fermentation, focusing on process optimization, biotechnological applications, and future perspectives. A systematic search was conducted using the scientific literature databases Google Scholar, PubMed, Scopus, and Web of Science. Search terms included “*Chlorella vulgaris* fermentation”, “bioprocess engineering”, “strain improvement”, and “bioactive compound production”. Peer-reviewed research articles and review papers published in English were included. Data from the last two decades (2000–2024) were considered, with a particular emphasis on studies from 2020 to 2024 to reflect the most recent advancements in optimizing fermentation processes and improving *C. vulgaris* strains. To minimize redundancy, only the most recent articles were selected in cases where duplicated entries appeared across multiple databases. The inclusion criteria were studies that provided experimental data or comprehensive reviews on fermentation processes, strain improvement techniques, or bioactive compound production in *C. vulgaris*. Studies that did not focus on these areas or were non-experimental were excluded from the review. This review synthesizes current findings on strain improvement, enhancements in bioactive compound production, and scale-up technologies. Additionally, it explores the emerging industrial applications of fermented *C. vulgaris* in nutraceuticals, pharmaceuticals, biofuels, wastewater treatment, and functional foods, highlighting its growing relevance in sustainable and environmentally friendly biotechnological processes.

## 2. Strain Improvement

### 2.1. Genetic and Metabolic Engineering Approaches

Recent developments in genetic and metabolic engineering have significantly enhanced the industrial potential of *C. vulgaris*, particularly for producing bioactive compounds and improving overall yield. The implementation of innovative techniques, such as Clustered Regularly Interspaced Short Palindromic Repeats-associated protein 9 (CRISPR-Cas9) genome editing and chloroplast transformation, has been vital in creating strains with enhanced metabolic pathways and greater productivity.

#### 2.1.1. Genetic Engineering Techniques

CRISPR-based methods hold great promise for optimizing key biological functions. For instance, targeted gene edits can enhance carbon fixation and nutrient uptake capabilities, leading to more efficient biomass production and higher metabolite yields [11]. Such advancements enable *C. vulgaris* to thrive under varying environmental conditions, a critical factor for its large-scale industrial applications.

One ground-breaking approach is applying the CRISPR-Cas9 system in *C. vulgaris*, which allows for precise genetic modifications without relying on traditional antibiotic resistance markers. This technology has enabled the creation of auxotrophic mutants, which act as platforms for further genetic modifications, thereby improving the efficiency of strain development for industrial purposes [12]. Additionally, researchers have developed chloroplast expression vectors to co-express antimicrobial peptides. These advancements enhance the microalga’s capacity to produce bioactive compounds while increasing its resilience under diverse environmental conditions [13].

#### 2.1.2. Metabolic Engineering for Bioactive Compounds

Metabolic engineering has further expanded the potential of *C. vulgaris* for industrial purposes by optimizing the synthesis of high-value compounds such as proteins, lipids, and carotenoids. Researchers have successfully overexpressed key enzymes and redirected metabolic fluxes to boost the production of bioactive molecules like lutein and β-carotene, which are highly sought after in the pharmaceutical and nutraceutical sectors [14]. Moreover, metabolic profiling studies have revealed how carbon and nitrogen allocation among different *C. vulgaris* strains directly influence biomass composition. For instance, strategies such as nanoparticle-assisted mass transfer in cultures have significantly increased lipid production, highlighting the benefits of integrating metabolic engineering techniques with innovative cultivation methods [15].

#### 2.1.3. Environmental Adaptability

Chlorella vulgaris is known for its remarkable adaptability to a wide range of environmental stressors, which enhances its potential for industrial-scale applications. Research indicates that specific strains of *C. vulgaris* can maintain high productivity and biosynthetic activity under suboptimal conditions, such as low or high temperatures, high light intensity, and nutrient deprivation, making it an ideal candidate for cost-efficient large-scale cultivation [16,17].

One key factor in its environmental resilience is its metabolic flexibility, enabling *C. vulgaris* to adjust its metabolic pathways in response to stress. This adaptability ensures the continued synthesis of bioactive compounds and supports growth even in less-than-ideal conditions. For example, certain strains can tolerate temperatures up to 30 °C, and some are capable of thriving under nutrient-limited conditions, such as nitrogen deprivation, which triggers increased lipid accumulation [17,18]. These metabolic adjustments are often driven by the expression of stress-responsive genes that help the algae optimize the use of available resources for growth and metabolite production [17]. In addition to temperature and nutrient stress, *C. vulgaris* has shown the ability to adapt to various other environmental factors, which include the following:

1. Temperature: *C. vulgaris* demonstrates significant adaptability to temperature variations, with growth observed between 20 °C and 35 °C. Optimal growth is typically achieved around 25 °C to 30 °C, depending on strain and cultivation conditions [19,20,21]. Growth rates decrease above 30 °C, with cultures dying at 38 °C. Conversely, lipid production and oleic acid content are enhanced under specific suboptimal or stress temperatures, highlighting potential applications in nutraceutical production [22].
2. Light: Light quality, intensity, and photoperiod play a critical role in the growth, chemical composition, and product yield of *C. vulgaris*. Significant findings include the following:
  - Light Intensity: Optimal growth was observed at lower intensities (62.5 µmol photons m^−2^ s^−1^) under a 16:8 light–dark cycle. Excessive intensity (>100 µmol photons m^−2^ s^−1^) can inhibit cell growth due to halted cell division [23].
  - Light Quality: Specific wavelengths affect product profiles. For instance, red light boosts chlorophyll, while green light promotes fatty acid production [24,25].
  - Photoperiod: Extended light exposure increases cell density, but balance is crucial to minimize energy costs and optimize yield [23,26].
3. Salinity: The salinity levels to which *C. vulgaris* can adapt play a critical role in its growth and metabolite production. Major insights include:
  - Optimal Salinity: Moderate salinity (30%) supports growth and enhances stress-induced metabolite production [18].
  - High Salinity Stress: While elevated NaCl concentrations (>0.4 M) inhibit growth, they promote lipid accumulation, making *C. vulgaris* a candidate for lipid production under stress conditions [22,27].
  - Metabolite Production: β-carotene production increases with moderate salinity (0.1–0.3 M NaCl) but declines at higher concentrations [28].
4. pH: An optimal pH of 8.0 supports the growth and health of *C. vulgaris*, with slight variations (pH 7.5–8.0) being suitable for cadmium-contaminated cultures. Unfavourable pH levels can stress the microalgae, affecting nutrient absorption and metabolite production and causing calcium precipitation, which ultimately inhibits growth [19,29].

This environmental adaptability not only makes *C. vulgaris* a robust candidate for various industrial applications but also reduces its reliance on controlled cultivation environments. Consequently, *C. vulgaris* can be cultivated across diverse geographical locations and climates, contributing to the sustainability and scalability of its use for biofuel production, food, and other biotechnological applications. By leveraging genetic and metabolic engineering tools, researchers have further enhanced the stress tolerance of *C. vulgaris*. These advancements enable the development of strains that can thrive under challenging environmental conditions, further improving the economic viability of large-scale algal cultivation. Key findings related environmental adaptability are summarized in Table 1.

#### 2.1.4. Integration of Engineering Approaches

Integrating genetic and metabolic engineering strategies has proven particularly effective in optimizing the production efficiency and economic feasibility of *C. vulgaris*. The combined use of CRISPR-based technologies and traditional metabolic engineering has enhanced the biosynthetic pathways for producing bioactive compounds, such as lutein, β-carotene, and proteins, which are valuable for the nutraceutical and pharmaceutical industries [11,14].

This integrated approach not only improves existing metabolic pathways but also opens avenues for exploring novel biosynthetic routes. Such innovations contribute to the development of sustainable bioprocesses that can meet the growing industrial demand for biofuels, nutraceuticals, and pharmaceuticals [39].

**Figure 1:**
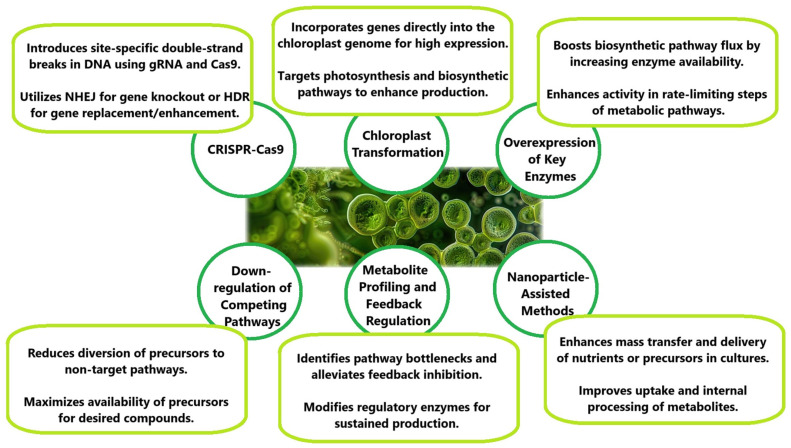
Overview of strain improvement techniques for enhancing metabolic pathways and production in *Chlorella vulgaris*.

Figure 1 demonstrates the primary strain improvement techniques for *C. vulgaris*, including CRISPR-Cas9, chloroplast transformation, metabolic engineering, and nanoparticle-assisted methods. These approaches enhance metabolic pathways, resilience, and production capabilities, optimizing the alga for industrial and biotechnological applications.

The strain improvement techniques illustrated in Figure 1 highlight the advancements in genetic and metabolic engineering for *C. vulgaris*. Each method contributes to enhancing the industrial potential of this microalga. For instance, CRISPR-Cas9 facilitates targeted genetic modifications, leading to increased carbon fixation and higher metabolite yields by knocking out competing pathways. Chloroplast transformation enables the high expression of transgenes, thus improving lipid biosynthesis and enhancing resilience through antimicrobial peptide production. The overexpression of key enzymes in biosynthetic pathways, such as those for carotenoids, significantly boosts the production of high-value compounds like lutein and β-carotene. The downregulation of competing pathways further redirects metabolic fluxes, maximizing lipid synthesis. Using metabolite profiling and feedback regulation, researchers identify and overcome bottlenecks in metabolic pathways, allowing the sustained production of bioactive compounds. Finally, nanoparticle-assisted methods enhance nutrient delivery and uptake in cultures, resulting in substantial increases in lipid content and overall biomass yield. These outcomes demonstrate how integrating these techniques can optimize *C. vulgaris* for diverse applications in biotechnology and industrial production.

### 2.2. Mutagenesis and Selection of Robust Strains

Mutagenesis continues to be a highly effective method for developing robust *C. vulgaris* strains, particularly those with enhanced resistance to environmental stressors and improved industrial applicability. UV-C mutagenesis, in particular, has been widely adopted to boost biomass productivity and lipid content. Research has demonstrated significant results, with one study reporting a 2.4-fold increase in lipid content in *C. vulgaris* mutants, showcasing the efficiency of UV-C radiation for enhancing strain performance in industrial contexts such as biofuel production [40,41]. Furthermore, UV-C mutagenesis has consistently been shown to increase total lipid content by up to 75%, making it a key technique for optimizing *C. vulgaris* strains for biofuel applications, where high lipid yields are crucial [40,41].

Chemical mutagenesis, particularly through agents like ethyl methanesulfonate (EMS), has proven to be an effective strategy for improving *C. vulgaris* for food applications. EMS has been employed to create chlorophyll-deficient mutants of *C. vulgaris*, which are particularly suitable for food and feed industries. These mutants demonstrate up to a 60% increase in protein content and reduced chlorophyll levels, making them ideal for products where taste and colour are critical considerations. The reduction in chlorophyll enhances the palatability of the biomass and aligns with consumer preferences for less green colouration in food products [14]. Moreover, EMS mutagenesis has been widely validated as a reliable method for inducing beneficial traits in various plant and algal species [11].

To further optimize these mutants, fluorescence-activated cell sorting (FACS) has been employed. This advanced screening technology enables the rapid evaluation of millions of cells, isolating strains with enhanced traits such as higher protein content. FACS has identified *C. vulgaris* mutants with up to 34% more protein content, significantly boosting their commercial viability for food and feed applications [39,42]. Its high-throughput capabilities ensure the efficient selection of mutants that not only meet nutritional requirements but also exhibit favourable growth characteristics [42].

The integration of EMS mutagenesis and FACS represents a powerful approach for improving *C. vulgaris* as a sustainable source of protein and bioactive compounds. These innovations support its use in a wide range of functional food, feed, and nutraceutical applications, addressing both nutritional and commercial demands [14,42].

By combining mutagenesis with advanced selection techniques, researchers are continuously developing *C. vulgaris* strains optimized for industrial processes. These robust strains ensure consistent production across multiple generations, making them suitable for large-scale fermentation and industrial applications such as biofuel production, nutraceuticals, and wastewater treatment [41].

### 2.3. Omics Approaches

Omics approaches—encompassing genomics, proteomics, and metabolomics—have become indispensable tools for improving *C. vulgaris* strains, particularly in enhancing yield and optimizing the production of valuable compounds. These methodologies provide a comprehensive understanding of metabolic pathways and regulatory networks, laying the groundwork for precise and targeted engineering strategies.

#### 2.3.1. Genomics

Genomics plays an important role in unravelling the genetic foundations of metabolic processes in *C. vulgaris*. For example, the genome assembly and annotation of the *C. vulgaris* 221/11P strain uncovered numerous previously uncharacterized genes, including those involved in fatty acid and lipid biosynthesis, which are essential for biofuel production [43]. Such insights enable researchers to identify key genes for overexpression or knockout, facilitating enhanced lipid accumulation and biomass productivity. Additionally, traits like oxidative stress resistance, identified through genomic analysis, can be exploited to develop strains with superior growth rates under suboptimal conditions, thereby improving overall productivity [44].

#### 2.3.2. Proteomics

The proteomic profiling of different *C. vulgaris* strains under varied environmental conditions has helped identify proteins upregulated in response to stress or nutrient changes, informing metabolic engineering efforts to optimize biosynthetic pathways for lipids and other valuable metabolites [45]. For instance, proteomic studies have illuminated critical bottlenecks in lipid biosynthesis pathways, guiding targeted interventions to boost lipid production in microalgae [46].

#### 2.3.3. Metabolomics

Metabolomics, which focuses on the small molecules within cells, provides a snapshot of the metabolic state of *C. vulgaris* under different cultivation conditions. Profiling metabolites under various growth scenarios enables researchers to identify key compounds associated with high biomass or lipid yields. Such insights are essential for fine-tuning cultivation strategies, including nutrient optimization and light intensity adjustments, to maximize productivity [45]. Furthermore, integrating metabolomic data with genomic and proteomic findings enables a systems biology approach, offering a holistic understanding of *C. vulgaris* metabolic networks and identifying novel targets for strain improvement [46].

To further illustrate the advancements achieved through omics approaches, Table 2 summarizes key studies that document improvements in biomass yield, lipid content, and metabolite production across *C. vulgaris* and related species. These findings highlight the integration of genomics, proteomics, and metabolomics with strain engineering and cultivation strategies to optimize productivity and compound synthesis for industrial applications.

## 3. Fermentation Techniques

### 3.1. Submerged vs. Solid-State Fermentation

Fermentation techniques play a key role in optimizing the yield and enhancing the bioactive compound production of *C. vulgaris*. The two main approaches are submerged fermentation (SMF) and solid-state fermentation (SSF), each offering unique benefits based on the target application and product outcome.

SMF involves cultivating *C. vulgaris* in a liquid medium, allowing precise control over factors such as pH, temperature, nutrient concentration, and aeration. SMF is particularly suited for large-scale biomass production, where consistent mixing and nutrient availability are crucial for maintaining yield and quality. The scalability and ease of process control make SMF ideal for industries prioritizing high biomass production with minimized contamination risks [54]. In industrial biofuel production, SMF has proven advantageous due to the increased lipid yield and controlled cultivation conditions necessary for efficient biomass and lipid recovery [55]. On the other hand, SSF is an innovative approach to enhancing the concentration and bioavailability of bioactive compounds in substrates such as *Chlorella vulgaris*. This method involves cultivating microorganisms on solid substrates with minimal free water, which often results in higher yields of targeted compounds like carotenoids and antioxidant peptides [56]. The technique is particularly suited for functional foods and nutraceutical applications, where maximizing bioactive compound production is more critical than large-scale biomass output.

The rich biochemical profile of *C. vulgaris*, which includes proteins, vitamins, and phytonutrients, makes it an ideal candidate for SSF processes aimed at producing high-value products. Moreover, its ability to serve as a substrate for co-fermentation with microorganisms further enhances its utility. For instance, fermentation with fungi such as *Aspergillus niger* can result in the production of organic acids like oxalic acid, demonstrating how *C. vulgaris* supports microbial metabolic activity while providing essential nutrients [57].

SSF also improves the overall nutritional profile of *C. vulgaris*, increasing the bioavailability of valuable compounds like the *Chlorella* growth factor. This compound is renowned for its health benefits, including immune system support and growth promotion [58]. Additionally, fermentation can produce beneficial metabolites that further enhance the functional properties of the end products.

Despite its advantages, SSF has limitations, such as increased contamination risks and the challenge of maintaining optimal moisture levels, which can hinder scalability [13]. However, these challenges have not overshadowed its potential for diverse applications, including sustainable energy production. Sequential processes involving anaerobic fermentation can yield biohydrogen while simultaneously generating bioactive compounds [59,60]. This dual functionality highlights *C. vulgaris*’s promise in advancing sustainability in food and energy systems.

Studies comparing SMF and SSF indicate that while SSF may produce higher levels of certain compounds, SMF is better suited for uniform, scalable biomass production. The choice between SMF and SSF often depends on the specific goals of the fermentation process, with SSF being preferable for concentrated bioactive compounds and SMF for efficient mass production.

### 3.2. Mixed Fermentation Processes

Mixed fermentation, involving multiple microorganisms, has emerged as an effective approach to enhance the nutritional profile and functional properties of *C. vulgaris*-based products. Co-fermentation with yeasts like *Rhodotorula glutinis* has shown improved lipid yields and carbon conversion efficiency, making it ideal for biofuel production. This co-culture approach allows for the simultaneous production of biofuels and wastewater treatment, as it enhances nutrient removal from wastewater while yielding a valuable lipid-rich biomass [54].

In functional food applications, mixed fermentation with lactic acid bacteria such as *Lactobacillus fermentum* has proven beneficial. When used in soy-based fermented beverages, *C. vulgaris* and lactic acid bacteria synergize to enhance antioxidant activity and improve nutrient bioavailability. This process improves both the flavour and health properties of the final product, making it a suitable option for probiotic beverages and functional foods [55].

Overall, mixed fermentation techniques are highly valuable for improving the sensory and nutritional qualities of *C. vulgaris*-based products. By combining multiple microbial activities, mixed fermentation can increase nutrient bioavailability, improve flavour profiles, and enhance stability, broadening the commercial applications of *C. vulgaris* in both biofuel and food industries.

### 3.3. Adaptability to Carbon Sources in Heterotrophic Cultivation

The cultivation of *C. vulgaris* in controlled fermentation tanks has gained considerable attention for its potential in high biomass production and the flexibility to utilize diverse carbon sources. The research underscores its adaptability to different carbon substrates, particularly under heterotrophic cultivation, which has shown promising outcomes for small-scale applications.

Heterotrophic cultivation enables *C. vulgaris* to metabolize organic carbon sources, resulting in enhanced growth rates and biomass yields compared to autotrophic methods. For example, Wu et al. [61] demonstrated that using sweet sorghum extract as a carbon source significantly increased carbon conversion efficiency and facilitated the production of value-added compounds, achieving higher biomass density and reduced culture periods in controlled fermentation systems. Similarly, substantial improvements in biomass and lipid productivity under heterotrophic conditions, highlighting the effectiveness of this approach for optimizing yields, have been reported [62]. Additionally, Silva and Fonseca [63] confirmed the capability of *C. vulgaris* to utilize various carbon sources, including glucose, fructose, and glycerol, to support robust growth under these conditions. Further evidence of *C. vulgaris’* adaptability to diverse carbon sources comes from studies on carbon-to-nitrogen (C:N) ratios. Glucose has been identified as the most effective organic carbon source, with an optimal C:N ratio of 18:1 yielding maximum growth rates [64]. This flexibility is particularly valuable for developing cost-effective and sustainable cultivation strategies, such as leveraging agricultural by-products or waste streams as carbon sources [65].

The cultivation mode also influences the nutritional composition of *C. vulgaris*. For instance, Sajadian et al. [66] emphasized the benefits of heterotrophic growth for lipid accumulation, making it a preferred approach for biofuel production. The use of different carbon substrates enhances biomass output and impacts fatty acid profiles, which are critical for biofuel and nutraceutical applications [62].

## 4. Bioprocess Engineering and Optimization

### 4.1. Bioreactor Design and Scaling Up

Optimizing bioreactor design is crucial for maximizing the production efficiency of *C. vulgaris*, especially for large-scale operations. Recent advances have focused on enhancing light penetration, gas exchange, and nutrient distribution to support high-density cultures. Flat-panel and tubular photobioreactors (PBRs) have shown particular promise, as these designs improve light exposure and photosynthetic efficiency, enabling more consistent biomass growth. For instance, studies have demonstrated that flat-panel bioreactors equipped with LED lighting and carbon dioxide control systems can significantly boost biomass yield in both laboratory and pilot-plant setups [67].

Airlift bioreactors are also widely used for *C. vulgaris* cultivation due to their low shear forces, which minimize cell damage and maintain culture viability over long periods. In a recent study, bubbling column designs proved superior for *C. vulgaris* by achieving the high biomass and lipid yields necessary for biofuel production, showing distinct advantages over stirred-tank reactors, which can cause cellular stress under continuous mixing [68]. Scaling up these bioreactors requires careful attention to light distribution and gas exchange efficiency, as demonstrated in large pilot-scale studies where optimal carbon dioxide levels and temperature control enhanced overall productivity [69].

### 4.2. Key Fermentation Parameters

The successful fermentation of *C. vulgaris* depends on the precise control of parameters such as pH, temperature, nutrient supply, and aeration. Maintaining optimal pH (around 7.5–8.0) and a temperature of approximately 25–30 °C is critical for maximizing biomass yield and bioactive compound production. Studies indicate that consistent aeration and balanced stirring are essential to avoid nutrient depletion and localized hypoxia, which can hinder cell growth. For example, optimized aeration in tubular PBRs led to significantly higher biomass output and lipid production rates, important for biofuel applications [67].

### 4.3. Monitoring Technologies

Advanced monitoring technologies are now essential in *C. vulgaris* fermentation, providing real-time data on critical parameters like pH, dissolved oxygen, and light intensity. The integration of sensor technologies with automated systems has allowed bioreactors to adjust environmental conditions dynamically. For example, artificial neural networks (ANNs) linked to bioreactor sensors can predict and adjust factors like light exposure and nutrient levels, ensuring optimal growth and maximizing compound synthesis [67]. Such technologies enhance consistency and efficiency, especially in large-scale operations, by minimizing human intervention and reducing the risk of contamination.

## 5. Downstream Processing

### 5.1. Separation and Purification of Bioactive Compounds

The efficient extraction and purification of bioactive compounds, such as proteins, chlorophylls and carotenoids, are essential for the industrial applications of *C. vulgaris*. Ultrasound-assisted extraction (UAE) has emerged as a particularly effective technique for this purpose. UAE utilizes high-frequency sound waves to disrupt *C. vulgaris* cell walls, significantly enhancing the release of bioactive compounds without compromising their structure. This approach is especially useful for extracting heat-sensitive compounds, preserving their bioactivity, and improving yield. Studies have shown that UAE increases the extraction rates of antioxidants and chlorophyll compared to traditional methods, making it well suited for applications in functional foods and nutraceuticals [70,71].

Microwave-assisted extraction (MAE) is another promising technique, producing high concentrations of chlorophyll and carotenoids with minimal processing time. MAE allows precise control over temperature and duration, which can enhance the purity and yield of extracted compounds. Comparative studies indicate that MAE can outperform UAE in producing higher concentrations of specific compounds, such as lutein and β-carotene, making it suitable for industrial applications requiring concentrated bioactive compounds [70,72].

Supercritical fluid extraction (SFE) has also gained attention for its environmentally friendly and efficient extraction capabilities. Using supercritical carbon dioxide as the solvent, SFE allows for the selective extraction of lipophilic compounds from *C. vulgaris*, achieving high-purity extracts suitable for food, cosmetics, and pharmaceuticals. This method is particularly valuable for extracting carotenoids and fatty acids due to its low-temperature requirements and reduced solvent use, contributing to sustainable and scalable bioactive compound production [71].

Table 3 summarizes the key methods that have been employed for the extraction and purification of bioactive compounds in *C. vulgaris*. These techniques, though developed for various other plants and microalgae, offer promising potential for adaptation to *C. vulgaris*, paving the way for innovative applications in this microalga.

### 5.2. Process Integration for Bioethanol and Biopeptide Production

A biorefinery approach maximizes the utility of *C. vulgaris* biomass by generating multiple valuable products through an integrated process. For example, the enzymatic hydrolysis of *C. vulgaris* biomass releases fermentable sugars, achieving a glucose yield of 90.4% from its carbohydrate content [78]. These sugars can then be fermented to produce bioethanol, with yields reaching up to 92.3% of the theoretical maximum using simultaneous saccharification and fermentation. In parallel, protein hydrolysis generates bioactive peptides with antioxidant and antihypertensive properties, which are suitable for nutraceutical and functional food applications [72]. Under optimal enzymatic hydrolysis conditions, extracts can contain up to 45% protein, with antioxidant activity of 1035 μmol TE/g protein and an IC50 for ACE inhibition of 286 μg protein/mL [71]. Additionally, these peptide hydrolysates have demonstrated antidiabetic potential, exhibiting 31% α-glucosidase inhibition [71].

This integrated approach improves the economic viability of *C. vulgaris* processing, allowing for the simultaneous production of biofuels and high-value nutraceutical ingredients. Studies show that such biorefinery setups yield bioethanol efficiently while producing bioactive peptides with notable health benefits, enhancing the overall sustainability and commercial appeal of *C. vulgaris* [68,71].

## 6. Applications of Fermented *Chlorella vulgaris*

### 6.1. Functional Food Ingredients

Fermented *C. vulgaris* has gained significant attention as a functional food ingredient due to its rich composition of bioactive compounds, including antioxidants, peptides, and polyphenols. The fermentation process enhances the bioavailability and concentration of these nutrients, making *C. vulgaris* a valuable addition to health-promoting foods. Fermentation with lactic acid bacteria (LAB) such as *Lactobacillus fermentum* and *L. rhamnosus* in soy-based beverages has been shown to boost antioxidant capacity, increase polyphenol content, and promote gut health by introducing beneficial probiotics. This process also improves protein digestibility and releases bioactive peptides with antioxidant and antimicrobial properties, further enhancing the drink’s overall nutritional profile [55]. Fermentation has demonstrated several advantages for *C. vulgaris* in food products [79]:

1. **Increased Antioxidant Activity:** Beverages supplemented with *C. vulgaris* extracts show higher levels of total polyphenols, flavonoids, and antioxidant activity compared to controls.
2. **Enhanced Probiotic Potential:** Incorporating *C. vulgaris* into fermented dairy products like yoghurt, cheese, and kefir increase lactic acid bacteria concentrations.
3. **Improved Nutrient Availability:** The fermentation process enhances the bioavailability of nutrients such as vitamins, minerals, and amino acids, which serve as supplementary nutrition for the microorganisms involved.
4. **Sensory Improvements:** Sensory evaluations reveal favourable impacts on aroma scores in fermented beverages containing *C. vulgaris* extract.

The mechanisms by which *C. vulgaris* enhances microbial growth during fermentation are multifaceted. The extract is rich in vitamins, minerals, amino acids, and growth factors, which support the growth and metabolic functions of fermenting microorganisms. Additionally, the antioxidant properties of *C. vulgaris*, particularly from chlorophyll and carotenoids, protect cells from oxidative stress, improving both microbial viability and fermentation efficiency [79].

These functional benefits position fermented *C. vulgaris* as an ideal ingredient for various food products, including protein supplements, beverages, and fermented snacks [70,80]. Its incorporation not only enhances nutritional value but also contributes to the development of novel functional foods with potential health-promoting properties.

### 6.2. Pharmaceutical and Cosmetic Applications

In the pharmaceutical sector, the bioactive compounds of *C. vulgaris* demonstrate significant therapeutic potential. Fermentation processes release bioactive peptides with immunomodulatory and anti-inflammatory effects, which are valuable for immune health and reducing oxidative stress. These peptides derived from fermented *C. vulgaris* have shown efficacy in modulating immune cell activity and cytokine production, making them promising candidates for nutraceuticals and functional supplements aimed at immune support [72].

In cosmetics, the potent antioxidant properties of *C. vulgaris* help combat oxidative stress, a major contributor to skin ageing. Its peptides and antioxidants can be used in skincare formulations to protect skin cells, provide anti-ageing benefits, as well as to prevent microbial contamination in cosmetic products [71].

Quantitative data support the pharmaceutical applications of fermented *C. vulgaris*. A recent study by Martinez-Ruiz et al. [81] found that *C. vulgaris* extract inhibited elastase activity by 90% at a concentration of 100 μg/mL, demonstrating strong anti-ageing potential, and another study by Morais et al. [82] showed that fermented *C. vulgaris* extract exhibited significant antioxidant activity, with an IC50 value of 52.28 μg/mL for DPPH radical scavenging.

Specific cosmetic applications include the use of *C. vulgaris* extracts in anti-ageing creams, moisturizers, and sunscreens [81], as well as the incorporation of *C. vulgaris*-derived pigments like carotenoids in makeup products [83] and the development of natural preservatives for cosmetic formulations [81].

### 6.3. Sustainability Aspects

*C. vulgaris* is recognized for its environmental benefits, especially in sustainable production practises. As a microalga, it captures carbon dioxide efficiently, converting it into high-nutrient biomass through photosynthesis. This capability not only helps reduce greenhouse gas emissions but also supports the development of eco-friendly food and bioactive compound production [80]. Additionally, integrating *C. vulgaris* into biorefinery processes allows for the production of bioethanol alongside bioactive compounds, maximizing resource use and economic efficiency. This dual-production approach maximizes resource utilization and enhances economic efficiency, generating renewable fuel while also yielding valuable nutraceutical ingredients [68]. Furthermore, *C. vulgaris* has shown potential in wastewater treatment by absorbing excess nutrients and contaminants, highlighting its role as an eco-friendly biotechnological solution [6].

Quantitative data further underscores the sustainability potential of *C. vulgaris*. Microalgae can fix 1.88 kg of CO_2_ per kg of algal biomass produced, with variations according to each algae genera, contributing to the reduction in greenhouse gas emissions [84]. In wastewater treatment, *C. vulgaris* has achieved remarkable removal efficiencies of up to 90% for nitrogen and 70% for phosphorus, demonstrating its effectiveness in environmental remediation [85]. Additionally, bioethanol production from *C. vulgaris* can yield up to 0.23 g of ethanol per g of biomass, highlighting its potential as a renewable and sustainable fuel source [82].

Table 4 provides an overview of the main applications of fermented *C. vulgaris*, organized across the following four key sectors: functional food ingredients, pharmaceuticals, cosmetics, and sustainability. Each sector is highlighted with its specific benefits, including the improved bioavailability of bioactive compounds, potent antioxidant properties, and environmental contributions such as carbon capture. This summary underscores *C. vulgaris*’s versatility and its rising significance in diverse industries, from health-focused products to eco-friendly solutions. Additionally, the table includes references to foundational studies supporting each application area, giving a comprehensive view of the scientific basis for *C. vulgaris*’s benefits and commercial relevance.

Incorporating *C. vulgaris* into the food industry is gaining momentum, particularly in the realms of nutraceuticals and functional foods. Known for its high protein content (51–58% of its biomass), essential fatty acids, vitamins, minerals, and bioactive compounds [86], *C. vulgaris* holds immense potential as a natural and sustainable ingredient. Its antioxidant, anti-inflammatory, and anti-microbial properties enhance the nutritional value of food products and contribute to improved shelf life and sensory attributes.

The microalga’s bioactive compounds, including carotenoids and polyunsaturated fatty acids, play a crucial role in these protective mechanisms, making it a valuable ingredient in functional foods aimed at disease prevention [87]. Additionally, *C. vulgaris* has shown the ability to modulate lipid profiles and enhance metabolic health [88], further supporting its potential as a natural bioactive agent in food products. These features, coupled with its capacity to address health concerns such as obesity, inflammation, and oxidative stress [89], position *C. vulgaris* as a transformative ingredient in the development of innovative, health-oriented food applications. Emerging research and industrial efforts underscore its versatility in areas such as meat fortification, functional beverages, and natural food colourants, paving the way for its integration into diverse food systems. Table 5 complements the information by providing a focused overview of how *C. vulgaris* can be applied in the food and nutraceutical industries, highlighting specific applications, and their associated benefits.

## 7. Future Perspectives and Challenges

### 7.1. Large-Scale Production Challenges

Scaling up *C. vulgaris* cultivation faces both technical and economic hurdles, particularly in optimizing bioreactor design and maintaining environmental control. Key challenges include ensuring adequate light distribution in high-density cultures and managing nutrient flow, pH, and temperature in large-scale photobioreactors. While designs like flat-plate and tubular photobioreactors improve light penetration and carbon dioxide absorption, transitioning from laboratory to industrial scales introduces issues such as contamination, fouling, and increased energy costs. These operational expenses and the complexity of maintaining consistent environmental conditions, especially in outdoor systems, underscore the need for cost-effective solutions [68,69].

### 7.2. Regulatory and Biosafety Considerations

The growing commercial use of *C. vulgaris* also demands updated regulatory frameworks, particularly regarding genetically modified strains. Biosafety concerns, including risks of environmental release and horizontal gene transfer, require stringent testing, especially in regions with strict policies like the EU. Harmonizing international standards would support the safer deployment of genetically modified *C. vulgaris* while encouraging technological innovation [94].

### 7.3. Potential Research Directions

Future research could focus on strain optimization through genetic and metabolic engineering to develop *C. vulgaris* strains with enhanced resistance to environmental stress and improved growth rates. CRISPR and other genetic editing tools offer promising avenues for enhancing nutrient composition, especially for biofuel applications where carbohydrate and lipid accumulation are crucial. Additionally, integrating *C. vulgaris* cultivation with biorefinery models and wastewater treatment systems could promote sustainable production, reducing costs and ecological impacts by utilizing waste resources effectively [67,71].

## 8. Conclusions

The fermentation of *C. vulgaris* has proven to be a transformative technique for enhancing the bioavailability and efficacy of its bioactive compounds, such as chlorophylls, carotenoids, and peptides. Advances in strain improvement, including genetic and metabolic engineering, have led to optimized *C. vulgaris* strains with increased yields and enhanced bioactive properties. While laboratory-scale studies have shown promising results, scaling up these techniques for industrial production remains a challenge. The comparative analysis of fermentation methods has shown that techniques like submerged and solid-state fermentation each offer unique advantages, particularly in optimizing nutrient availability and compound extraction. However, achieving consistent and high-yield fermentation parameters across large-scale operations is a key bottleneck that requires further optimization.

Innovations in bioreactor design, such as flat-panel and tubular photobioreactors, have advanced the scalability of *C. vulgaris* cultivation, although issues such as maintaining light intensity, temperature control, and oxygenation still need to be addressed for large-scale applications. Biorefinery models have maximized economic and environmental efficiencies, but the integration of these processes into industrial settings requires overcoming challenges related to process control, cost-effectiveness, and waste management.

Applications of fermented *C. vulgaris* now span across the functional food, pharmaceutical, and cosmetic industries, driven by its versatile bioactive profile. However, transitioning these applications from laboratory experiments to large-scale production remains a critical challenge, particularly in terms of product consistency and regulatory compliance. Additionally, *C. vulgaris*’s potential role in carbon sequestration and wastewater treatment underscores its value as an eco-friendly biotechnological resource, though practical implementation on a global scale would require overcoming regulatory hurdles and establishing cost-effective infrastructure

However, challenges remain, especially in large-scale production, addressing biosafety regulations and achieving consistent fermentation parameters for reliable yields.

Prospects for *C. vulgaris* commercialization are immense, particularly with ongoing research in strain optimization, bioprocess engineering, and sustainable integration. Continued advancements in these areas, combined with applications in carbon capture and waste management, position *C. vulgaris* as a pivotal resource in tackling global environmental and food security challenges. As genetic and bioprocess technologies evolve, *C. vulgaris* is well poised to become an essential asset across biotechnology fields, but its widespread industrial application will depend on overcoming the critical barriers to scalability and process optimization.

## Figures and Tables

**Table 1:** Summary of environmental factors influencing growth and metabolite production in *Chlorella vulgaris* and other algae.

Effect on *Chlorella vulgaris* and Other Algae		References
**Temperature Effect**		
**Temperature Range**		
20–35 °C	General growth range	[19]
~25 °C	Optimal growth temperature for most strains	[19]
30 °C	The highest biomass production observed	[20,21]
Below 16 °C	Limited cell growth	[30]
>35 °C	Growth inhibition; culture death at 38 °C	[20,21]
Decrease from 30 °C to 25 °C	Lipid production increased to 14.7%	[22]
38 °C	Oleic acid content increased to 34%	[22]
**Light Effect**		
**Light Conditions**		
Blue light	Enhanced biomass production (up to 1.3 g/L)	[19]
Yellow, red, white light	Faster log-phase growth compared to blue, green, and purple light	[25]
Green light	Increased hexadecatrienoic acid (C16:3) and α-linolenic acid (C18:3)	[25]
Red light	Enhanced chlorophyll production due to red light absorption	[24]
Dark conditions	Higher polyunsaturated fatty acid content (e.g., docosahexaenoic acid)	[31,32]
62.5 µmol photons m^−2^ s^−1^, 16:8 L:D	Maximum biomass (2.05 ± 0.1 g/L)	[23]
100 µmol photons m^−2^ s^−1^	Decreased biomass compared to lower intensity	[23]
100–200 µmol photons m^−2^ s^−1^	Common light intensity for cultivation	[33]
**Salinity Effect**		
**Salinity Conditions**		
Up to 3.5% NaCl	Tolerable salinity for freshwater strains	[34]
Moderate salinity (30‰)	Optimal for growth- and stress-induced metabolite production	[18]
<0.5 M NaCl	Maximum salinity adaptation	[35]
0.1–0.3 M NaCl	Increased β-carotene production	[28]
0.4 M NaCl	Decreased β-carotene production	[28]
0.5 M NaCl	Reduced growth but increased lipid accumulation due to oxidative stress	[22,27]
**pH Effect**		
**pH Conditions**		
pH 8.0	Ideal for growth and overall health of *C. vulgaris*	[19]
pH 7.5–8.0 (Cadmium culture)	Suitable for growth in cadmium-contaminated environments	[36]
pH 10.0–10.5	Optimal for normal cultivation	[37]
pH 3.0–6.2 (Acidic)	Slower growth due to acidic conditions	[36]
pH 8.3–9.0 (Alkaline)	Slower growth under alkaline conditions	[36]
Unfavorable pH levels	Can cause calcium precipitation, affecting nutrient absorption	[38]
Constant pH adjustment	Enhances growth and prevents contamination	[37]

**Table 2:** Summary of key studies utilizing omics approaches to improve biomass yield, lipid content, and metabolite production in *Chlorella vulgaris* and related species.

Key Findings	Reported Improvements	References
**Biomass Yield**		
Increased CO_2_ (1–8%) optimized biomass productivity, but higher concentrations reduced yield	Enhanced biomass productivity under optimal CO_2_ levels	[47]
Overexpression of AtLEC1 transcription factor improved lipid accumulation, indirectly increasing biomass yield in oleaginous strains	Significant biomass yield improvement correlated with lipid increases	[48]
**Lipid Content**		
Nitrogen deprivation increased lipid content up to 70% of dry weight	Dramatic increase in lipid content under nutrient deprivation	[49]
Nitrogen deprivation shifted fatty acid profiles from polyunsaturated to saturated/monounsaturated, improving suitability for biodiesel	Enhanced lipid composition for biofuel applications	[50]
Genomic analysis identified 2285 genes related to lipid metabolism, enabling targeted genetic modifications for improved lipid synthesis	Genetic targets for lipid content enhancement identified	[43]
**Metabolite Production**		
Mixotrophic growth with glucose and light improved biomass growth and lipid accumulation	Significant enhancement in metabolite production and biomass growth	[51]
Elevated CO_2_ levels enhanced carbon utilization, lipid accumulation, and energy production	Improved metabolite production through carbon optimization	[52]
**Overall Productivity**		
Nutrient optimization significantly enhanced specific growth rates and biomass productivity, crucial for commercial applications	Increased specific growth rates and overall productivity under optimized growth conditions	[53]

**Table 3:** Overview of extraction and purification methods for bioactive compounds reported in various plants and microalgae, with potential applicability to *Chlorella vulgaris*.

Method	Extracted Compounds	Identification Techniques	Quantitative Findings	References
**Ultrasound-** **Assisted** **Extraction**	Proteins, chlorophylls, antioxidants	High-Performance Liquid Chromatography (HPLC)	Chlorophyll yield increased by 30% compared to conventional methods; Antioxidant extraction rates improved by 25–40%; a A 2.5-fold increase in total phenolic content at 40 kHz for 30 min compared to maceration	[70,71,73,74]
**Microwave-** **Assisted** **Extraction**	Chlorophyll, carotenoids (e.g., lutein, β-carotene	UV-Vis Spectroscopy, HPLC	Lutein yield of 4.7 mg/g (1.8× higher than UAE); β-carotene extraction of 2.3 mg/g (40% improvement over conventional methods); Reduced extraction time by 75% compared to traditional techniques	[70,72,75,76]
**Supercritical Fluid** **Extraction**	Carotenoids, fatty acids	Gas Chromatography–Mass Spectrometry (GC-MS)	Total carotenoid extraction of 18.5 mg/g at 60 °C and 350 bar (30% higher than solvent extraction); Fatty acid efficiency increased by 22%; Solvent use reduced by 90%	[71,76,77]

**Table 4:** Major areas of applications of fermented *Chlorella vulgaris* and related key benefits.

Application Area	Key Benefits	References
**Functional Food Ingredients**		
Inclusion in protein supplements, beverages, snacks	Enhances the bioavailability of nutrients, increases antioxidant activity, and supports gut health through probiotics	[55,70,80]
	**Pharmaceuticals**	
Nutraceuticals, immune support supplements	Provides bioactive peptides with immunomodulatory and anti-inflammatory properties; reduces oxidative stress	[71,72]
	**Cosmetics**	
Anti-ageing creams, antioxidant serums	Potent antioxidant and antimicrobial properties; protects skin from oxidative damage and enhances cell health	[68,71]
	**Sustainability**	
Biofuel production, wastewater treatment	Carbon sequestration, eco-friendly bioethanol production, and nutrient recovery in wastewater treatment	[68,80]

**Table 5:** Emerging industrial applications of fermented *Chlorella vulgaris* in functional foods and nutraceuticals.

Application	Description	Benefits	Reference
**Meat Products** **(e.g., Burgers)**	*C. vulgaris* incorporated into meat products to enhance their nutritional composition	Improved protein content, reduced fat levels, antioxidant, and antimicrobial effects	[90]
**Baked Goods** **(e.g., Cookies)**	Used as a natural colourant and nutrient enhancer in dough formulations	Enhanced sensory properties, increased fibre and nutrient density, and improved rheological behaviour	[91]
**Functional Beverages**	Development of alcoholic and non-alcoholic beverages enriched with *C. vulgaris* extracts	Antioxidant properties and health benefits, appeal to wellness-conscious consumers.	[92]
**Food Preservation**	Application of *C. vulgaris* for extending shelf life in perishable foods	Antimicrobial and antioxidant activities to prevent spoilage and maintain freshness	[90]
**Nutraceutical Supplements**	Production of capsules or powders containing fermented *C. vulgaris* biomass	Anti-inflammatory, anti-obesity, and antioxidant effects; support for lipid profile modulation	[93]
**Aquaculture Feed** **Enhancement**	Inclusion of *C. vulgaris* in fish feed formulations	Improved growth, immune response in aquatic species, and sustainability in aquaculture practises	[1]

## Data Availability

No new data were created or analyzed in this study. Data sharing is not applicable to this article.
